# PDE5 inhibitor drugs for use in dementia

**DOI:** 10.1002/trc2.12412

**Published:** 2023-09-25

**Authors:** Atticus H. Hainsworth, Ottavio Arancio, Fanny M. Elahi, Jeremy D. Isaacs, Feixiong Cheng

**Affiliations:** ^1^ Molecular & Clinical Sciences Research Institute St George's University of London London UK; ^2^ Department of Neurology St George's University Hospitals NHS Foundation Trust London UK; ^3^ Department of Pathology and Cell Biology Taub Institute for Research on Alzheimer's Disease and the Aging Brain Department of Medicine Columbia University New York New York USA; ^4^ Departments of Neurology and Neuroscience Ronald M. Loeb Center for Alzheimer's Disease Friedman Brain Institute Icahn School of Medicine at Mount Sinai New York New York USA; ^5^ Genomic Medicine Institute Lerner Research Institute Cleveland Clinic Cleveland Ohio USA; ^6^ Department of Molecular Medicine Cleveland Clinic Lerner College of Medicine Case Western Reserve University Cleveland Ohio USA

**Keywords:** Alzheimer's disease, ardenafil, clinical trials, dementia, drugs, PDE5 inhibitors, repurposing, sildenafil, tadalafil, vascular contributions to cognitive impairment and dementia

## Abstract

Alzheimer's disease and related dementias (ADRD) remain a major health‐care challenge with few licensed medications. Repurposing existing drugs may afford prevention and treatment. Phosphodiesterase‐5 (PDE5) is widely expressed in vascular myocytes, neurons, and glia. Potent, selective, Food and Drug Administration–approved PDE5 inhibitors are already in clinical use (sildenafil, vardenafil, tadalafil) as vasodilators in erectile dysfunction and pulmonary arterial hypertension. Animal data indicate cognitive benefits of PDE5 inhibitors. In humans, real‐world patient data suggest that sildenafil and vardenafil are associated with reduced dementia risk. While a recent clinical trial of acute tadalafil on cerebral blood flow was neutral, there may be chronic actions of PDE5 inhibition on cerebrovascular or synaptic function. We provide a perspective on the potential utility of PDE5 inhibitors for ADRD. We conclude that further prospective clinical trials with PDE5 inhibitors are warranted. The choice of drug will depend on brain penetration, tolerability in older people, half‐life, and off‐target effects.

**HIGHLIGHTS:**

- Potent phosphodiesterase‐5 (PDE5) inhibitors are in clinical use as vasodilators.
- In animals PDE5 inhibitors enhance synaptic function and cognitive ability.
- In humans the PDE5 inhibitor sildenafil is associated with reduced risk of Alzheimer's disease.
- Licensed PDE5 inhibitors have potential for repurposing in dementia.
- Prospective clinical trials of PDE5 inhibitors are warranted.

## 1 INTRODUCTION

### 1.1 Dementia and current treatments

Age‐associated neurodegenerative disorders, including Alzheimer's disease (AD) and AD‐related dementias (ADRD), represent a major global health‐care challenge with few treatment options. The significant contribution of vascular disease to ADRD, alongside or interacting with neurodegenerative pathologies, is embodied in the concept of vascular contributions to cognitive impairment and dementia (VCID).^1^, ^2^ Currently no drugs are evidenced to specifically treat VCID.^3^, ^4^ Repurposing existing, approved drugs may yield new pharmaceutical treatment options for these prevalent pathologies and diminish the burden of disability.^5^

**FIGURE 1:**
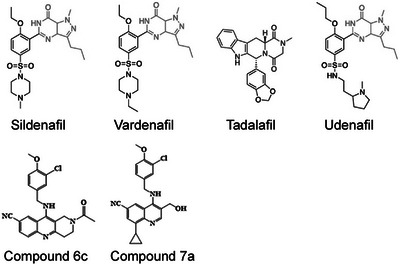
Chemical structures of licensed and novel phosphodiesterase‐5 inhibitor compounds.

Phosphodiesterase‐5 (PDE5) inhibitors (PDE5i) are widely prescribed for erectile dysfunction (ED) and pulmonary arterial hypertension (PAH). Their known mechanism in these diseases is to augment nitric oxide‐dependent vasodilation, by impeding PDE5‐mediated cyclic guanosine monophosphate (cGMP) breakdown in vascular myocytes.^6^ Three PDE5i are currently licensed by the US Food and Drug Administration (FDA) for clinical use, sildenafil (brand named Viagra^®^ or Revatio^®^), vardenafil (Levitra®), and tadalafil (Cialis®); see structures in Figure 1.

A recent study combined endophenotype‐based in silico network medicine discovery with real‐world patient data mining from insurance records to identify candidate drugs for repurposing in AD. The shortlist of 21 drugs included two PDE5i, sildenafil and vardenafil, with sildenafil identified as the overall best candidate.^7^ The hypothesized benefit from this study is congruent with pre‐clinical in vivo and in vitro data. Multiple research groups have reported that treatment with PDE5i ameliorated synaptic function in animal models, alongside improved behavioral performance in cognitive paradigms and biochemical markers of memory.^8^, ^9^, ^10^, ^11^, ^12^, ^13^, ^14^, ^15^, ^16^ PDE5i merit further investigation for possible use in dementia.^17^ Here we review the existing evidence base and discuss some key issues.

### 1.2 PDE5 and other phosphodiesterases

PDE5 belongs to one of the eleven subfamilies of phosphodiesterase (PDE) enzymes that are responsible for the degradation of two cyclic nucleotides, cGMP and cyclic adenosine monophosphate (cAMP). While PDE1, 2, 3, 10, and 11 degrade both cyclic nucleotides, PDE5, 6, and 9 degrade only cGMP, and PDE4, 7, and 8 degrade only cAMP.^6^ PDE5 is a critical component of a cascade of second messengers that starts with the release of nitric oxide (NO), activating soluble guanylyl cyclase that releases cGMP which, in turn, activates protein kinase G (PKG). This enzyme phosphorylates (among other targets) the transcription factor cyclic adenine monophosphate responsive element binding protein (CREB; so‐called NO/cGMP/PKG/CREB signaling pathway). Among other PDE family members, PDE5 is documented in brain tissue at mRNA and protein level.^18^, ^19^ PDE5 is present in human brain neurons^20^ and in vascular myocytes within subcortical white matter.^21^

## 2 PDE5 INHIBITORS

### 2.1 Existing licensed drugs and indications

Zaprinast was the first synthesized PDE5i. It is a bronchodilator in exercise‐associated asthma and induced smooth muscle relaxation and a NO/cGMP‐dependent relaxation of the corpus cavernosum. It predated the chemically related cGMP‐based derivative sildenafil,^22^ which was originally developed as an anti‐hypertensive drug, but ultimately was approved by the FDA for ED.^23^ Subsequently, new PDE5i agents (vardenafil, tadalafil, avanafil) received FDA approval for the treatment of ED. These drugs were followed by a new generation of PDE5i, lodenafil (Helleva), udenafil (Zydena), and mirodenafil (Mvix), that have not been approved by the FDA, but are available in Brazil and Korea for ED treatment.^24^

The three best known potent, selective PDE5i drugs are sildenafil, tadalafil, and vardenafil (for a pharmacological comparison, see Table 1). All three have low nanomolar IC_50_ for PDE5 (Table 1). Sildenafil has cross reactivity with PDE6 (IC_50_ ≈ 10 nM). Vardenafil likewise cross‐reacts with PDE6 in the low nanomolar range and, at much higher concentrations, with PDE1, 9, and 11 (Table 1). Tadalafil has interactions with PDE11, and at much higher concentrations, PDE6 (Table 1). The older, much less‐potent agents dipyridamole and zaprinast are also shown in Table 1 for comparison.

**TABLE 1:** Potency of well‐known PDE5 inhibitors. Potencies stated are IC50 values unless otherwise stated.

Drug name	Potency: PDE5i	Potency: other actions	References
Sildenafil	1–9 nM	PDE6: 10–40 nM	^8^, ^25^, ^26^, ^27^, ^28^
	K_D_ 3.1 nM		
Tadalafil	1–7 nM	PDE6: K_i_ 700 Nm PDE11: 10—300 nM	^8^, ^25^, ^26^, ^27^, ^29^, ^30^
	K_D_ 1.7 nM		
	K_i_ 1.9 nM		
Vardenafil	0.1‐1.0 nM	PDE1: 300 nM	^8^, ^25^, ^26^, ^29^
	K_D_ 0.32 nM	PDE6: K_i_ 0.3‐11 nM	
	K_i_ 2.3 nM	PDE9: 680 nM	
		PDE11: 240 nM	
Udenafil	6–8 nM	PDE 1: 870 nM	^31^
		PDE 6: 50 nM	
Dipyridamole	900–2000 nM	PDE6: 380–1000 nM	^27^, ^28^, ^30^
		PDE7: 1–2 μM	
		PDE10: 1 μM	
		PDE11: 800 nM	
Zaprinast	50 nM–2.5 μM^a^	PDE6: 150 nM	^27^, ^28^

^a^ Wide species and tissue dependence of zaprinast potency as a PDE5i.

Abbreviation: PDE, phosphodiesterase; PDE5i, phosphodiesterase‐5 inhibitor.

**RESEARCH IN CONTEXT**

1. **Systematic review**: The authors reviewed previous work and literature on phosphodiesterase‐5 (PDE5) inhibitor drugs for possible utility in dementia, based on their own expertise and literature searches (PubMed).
2. **Interpretation**: Animal studies from multiple laboratories, including our own, point to a cognitive enhancing action of PDE5 inhibitor drugs. This may be due to augmented nitric oxide signaling after PDE5 inhibition, leading to synaptic strengthening. Further, retrospective pharmaco‐epidemiologic analyses of insurance claims data for 7 million older people revealed that sildenafil use was associated with reduced risk of Alzheimer's disease.
3. **Future directions**: We propose that further prospective clinical trials with PDE5 inhibitor drugs are warranted. The test drug will require careful selection in terms of tolerability, brain penetration, half‐life, and possible off‐target effects.

### 2.2 Novel compounds with ideal central nervous system and blood–brain barrier‐penetrant profiles

The hypothesis that PDE5i may be AD/ADRD therapeutics derived from the involvement of the NO cascade in memory mechanisms.^9^ Following the initial publication of a proof‐of‐concept manuscript using sildenafil as a classical PDE5i,^9^ a series of studies was performed aiming at developing inhibitors with improved selectivity with respect to PDE5 versus other PDE isoforms. The rationale behind this approach was the need for reducing side effects in chronic conditions, such as ADRD, which generally affect an elderly population with likely comorbidities. Quinoline‐based, naphthyridine‐based, and 1H‐pyrroloquinolinone‐based PDE5i were proposed to have potential for chronic treatment of ADRD patients.^8^ The quinoline compound 7a (Figure 1) potently inhibited PDE5 (IC_50_ 0.27 nM) and readily crossed the blood–brain barrier (BBB). The compound rescued synaptic and memory defects in an in vivo mouse model of amyloid elevation,^8^ as well as after tau elevation.^11^ Importantly, compared to sildenafil, vardenafil, and tadalafil, 7a showed improved PDE5/PDE6 potency (IC_50_ = 339 nM for PDE6) and did not inhibit any of the other PDE isozymes. As the compound showed a low water solubility, a new scaffold was designed by locking the rotatable bonds of the hydroxymethyl group of the quinoline‐base compounds into a ring, which ultimately led to the design of compound 6c, a naphthyridine‐based and 1H‐pyrroloquinolinone‐based PDE5i.^32^ Compound 6c showed improved water solubility with respect to compound 7a, with excellent PDE5 potency and selectivity (IC_50_  =  0.056 nM for PDE5, 30.1 nM for PDE6), and 6 minute half‐life in human liver microsome stability test, indicating rapid metabolism of the compound by human microsomes. Most importantly, 6c ameliorated learning and memory deficits in a mouse model of amyloid elevation. In silico docking studies identified two plausible binding modes in the same pocket, which provided insights into the structural basis of PDE5i activity. Further optimization studies via a structure‐based approach may provide candidates for ADRD therapy.

## 3 PDE5I FOR POSSIBLE CLINICAL USE IN AD/ADRD?

### 3.1 Evidence from real‐world patient data

**FIGURE 2:**
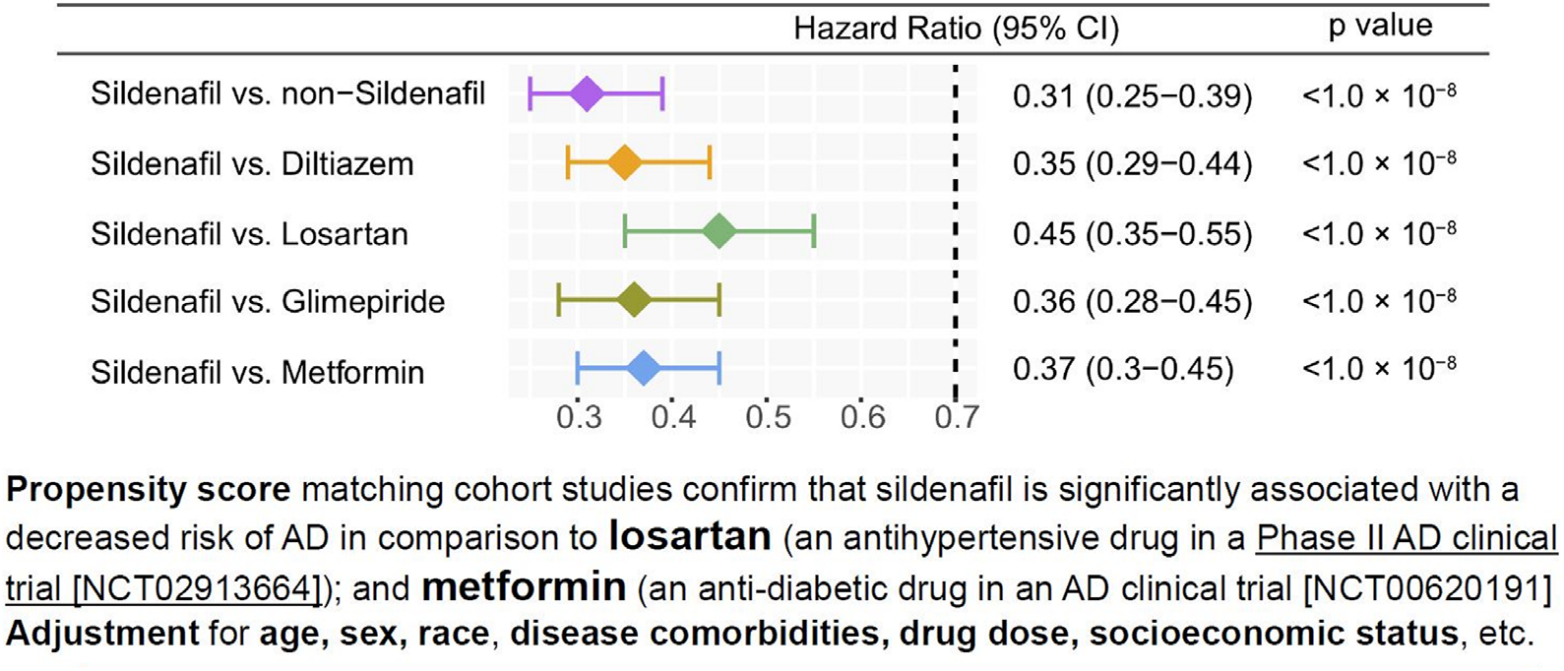
Hazard ratios and 95% confidence interval for sildenafil across five cohort studies: (1) sildenafil (*n* = 116,412) versus propensity score (PS)–matched control population (*n* = 465,648), (2) sildenafil versus diltiazem (*n* = 248,455), (3) sildenafil versus losartan (*n* = 2,036,012), (4) sildenafil versus glimepiride (an anti‐diabetic drug, *n* = 159,597), and (5) sildenafil versus metformin (*n* = 460,356). Using PS‐stratified survival analyses, non‐exposures and two comparators were matched to the exposures by adjusting age, sex, race, and comorbidities. From Fang et al.^7^ Reproduced with permission. AD, Alzheimer's disease; CI, confidence interval.

Based on amyloid and tau synergistic endophenotype findings, Fang et al., identified that sildenafil usage was significantly associated with ≈ 30% to 60% reduced risk of AD, using retrospective case–control pharmacoepidemiologic analyses of insurance claims data for 7.23 million older individuals.^7^ Propensity score–stratified analyses confirmed that sildenafil use was significantly associated with a decreased risk of AD across all four drug cohorts tested (diltiazem, glimepiride, losartan, and metformin, Figure 2) after adjusting age, sex, race, and disease comorbidities. In the same study, sildenafil increased neurite growth and decreases phospho‐tau expression (i.e., p‐tau181) in AD patient induced pluripotent stem cell (iPSC)–derived neuron models, supporting mechanistically its potential beneficial effect in AD.^7^

This study has several limitations.^7^ First, the association between sildenafil use and decreased incidence of AD does not establish causality or its direction, which requires further studies, including more real‐world patient data observations and clinical trials. Second, by lack of true active compactor design analysis, sildenafil may be more likely to be prescribed for wealthy individuals and increased wealth was associated with a reduced risk of developing AD. Propensity score–based analyses account only for measured differences in characteristics between exposure groups. In addition, the used propensity score approach may have been susceptible to unmitigated time‐related biases. The influence of unmeasured differences not available in insurance claims, such as frailty, blood pressure control, or glycemic control, cannot be determined and are likely to be important. Additional pharmacoepidemiologic studies with appropriate study designs are warranted in the future, using causal inference approaches.^33^

In contrast to the findings of Fang et al., a recent study by Desai et al. in PAH patients reported no significant association between initiation of PDE5i and risk of incident ADRD.^34^ The authors compared incidence of ADRD between patients taking PDE5i (sildenafil and tadalafil) to those taking an endothelin receptor antagonist (ERA), using a 1:1 propensity score–matching study. They found no significant association between ADRD incidence between PDE5i users and ERA users.^34^ In that study confounding by indication was mitigated using an active‐comparator, new‐user design in people with the indication.^34^ This design was a strength, ensuring that the treatment group and the comparator did not differ widely in most characteristics.

There are also potential limitations in the Desai et al. study.^34^ First, PAH is a rare disease, usually seen in adults under the age of 60, and carries a poor prognosis (35% survival at 3 years). PAH represents a limited population in the Medicare claims database. Hence there is a risk that the study may be under‐powered. A second, related concern is the duration of follow‐up of patients included in the analysis (approximately 6 months).^34^ This is a relatively short time window, unlikely to be sufficient for detecting cognitive differences in a dementia trial. Third, the doses of PDE5i agents in many patients were too low to achieve adequate brain concentrations to produce a potential central effect.^34^ Thus it seems premature to conclude that PDE5i are without efficacy for treating dementia.^34^

### 3.2 Neuronal or vascular site of action?

Current prescribing of PDE5i drugs is based on a vascular site of action. The enzyme is located within vascular myocytes and by degrading cGMP is a determinant of vasoconstrictor tone. PDE5i treatment preserves cGMP and promotes vasodilatation. In the context of brain vasculature, PDE5i action might be expected to dilate small penetrating arteries and thus to increase perfusion and metabolic health of downstream brain tissue. Alternatively, a neuronal site of action may be predicted. PDE5 is expressed in brain neurones.^20^ At the synapse, cGMP participates in the nitric oxide–dependent process of synaptic strengthening (an example being “long‐term potentiation”).^10^, ^35^ On this basis we hypothesize that brain‐penetrant PDE5i drugs have potential to augment NO‐driven synaptic strengthening and thus to improve memory function. Relevant studies in humans and in experimental animals are discussed in Section 4.

## 4 TESTING A PDE5I FOR POSSIBLE USE IN DEMENTIA

### 4.1 The PASTIS trial: testing tadalafil for use in VCID

We recently performed a phase II randomized clinical trial of the PDE5i tadalafil in older women and men with symptomatic small vessel disease,^36^ which is the major cause of vascular cognitive impairment.^37^ The Perfusion by Arterial Spin Labelling Following Single Dose Tadalafil in Small Vessel Disease (PASTIS) trial is registered at: https://clinicaltrials.gov/ct2/show/NCT02450253. We compared a single dose of tadalafil (20 mg) to placebo, the primary outcome measure being change in subcortical cerebral blood flow (CBF) measured by arterial spin labeling.^38^ Secondary outcome measures included change in cortical CBF, and a panel of neuropsychological assays of cognition (listed in the protocol^38^).

We selected tadalafil on the basis of long plasma half‐life (16 hours)^39^, ^40^ and evidence of brain penetration. Reports in experimental rodents and primates indicate that tadalafil crosses the BBB.^13^, ^41^, ^42^ Oral dosing of non‐human primates with 2.4 mg/kg tadalafil gave brain concentrations of 10 nM,^42^ well above the concentration for half maximal PDE5 inhibition. The tadalafil brain:plasma concentration ratios reported in animals are modest (0.10–0.12)^13^, ^41^ but appreciably greater than those reported for sildenafil (0.028–0.050).^41^, ^43^

**FIGURE 3:**
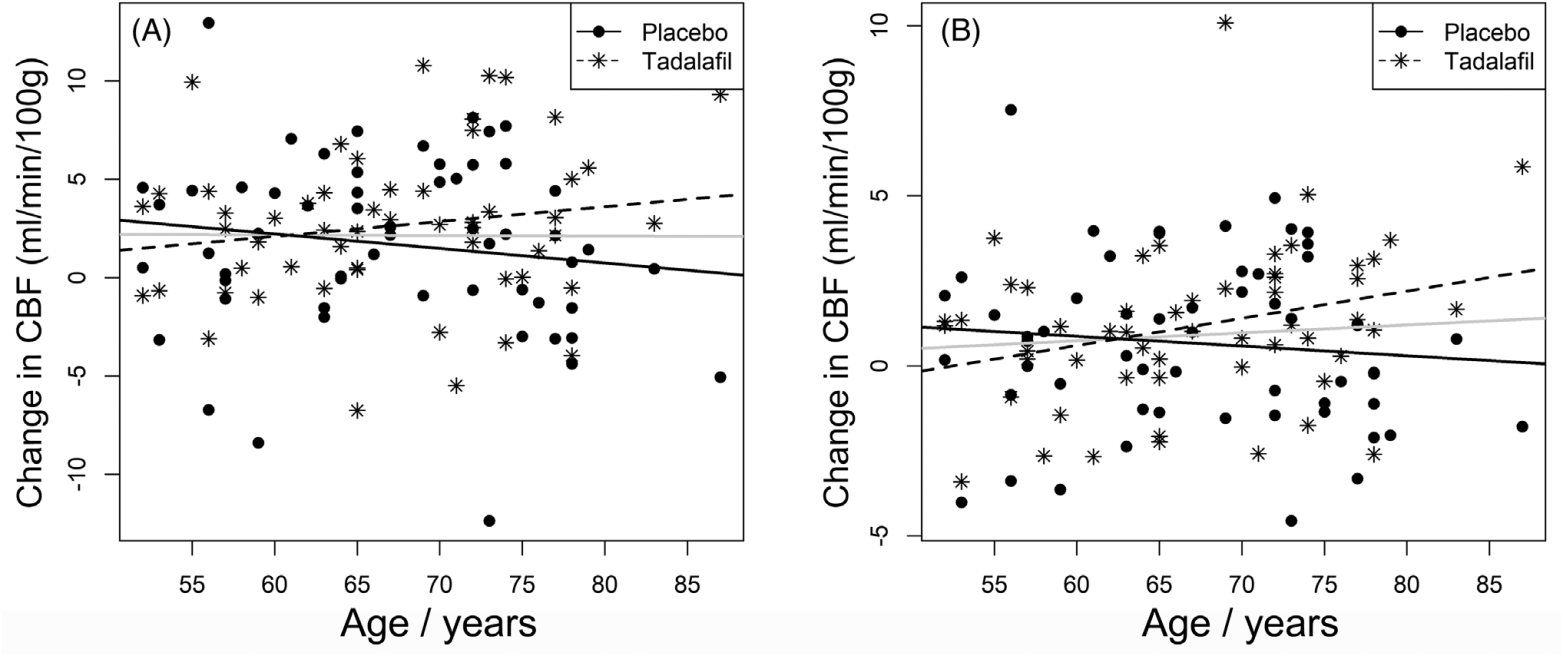
Change in cerebral blood flow (CBF) after placebo or tadalafil in older people with small vessel disease, as a function of age. Change in CBF in total gray matter (A), and normal‐appearing white matter (B). Lines of best fit are shown for placebo (solid line), or tadalafil (dashed line) or for all data points (gray line). Figure from Pauls et al.,^36^ reproduced with permission.

In the PASTIS trial we did not detect a difference between tadalafil and placebo with respect to CBF, despite a small but significant reduction in blood pressure after tadalafil.^36^ The sample size was *N* = 65, with 55 completing the protocol. Our a priori power calculation indicated a need for 54 participants to detect a 15% change in subcortical CBF. The largest effect size observed was a trend for increased blood flow within white matter hyperintensities, which are a radiological feature of cerebral small vessel disease (9.8% increase, *P* = 0.096).^44^ In post hoc analyses, we noticed a trend for increased brain blood flow in participants aged > 65 (Figure 3).^36^ No serious adverse events were observed.

### 4.2 PDE5i in ADRD

Two pilot studies showed potential benefits of sildenafil in treatment of AD. In one, a single dose of 50 mg sildenafil decreased spontaneous neural activity in the right hippocampus in 10 patients.^45^ In a second, a single dosage of 50 mg sildenafil increased cerebral metabolic rate of oxygen and CBF in 12 patients, and decreased cerebral vascular reactivity in 8 patients.^46^ It is challenging to interpret these data quantitatively, owing to the small cohorts and lack of control participants. Nevertheless, they offer intriguing pilot data.

### 4.3 Other clinical studies

Other studies have reported changes in CBF after PDE5i treatment in older people with brain disease^46^, ^47^, ^48^ though no clear message emerges. In a small series of 24 consecutive male patients with ED, a diverse pattern of regional CBF changes was apparent, 1 hour after a single oral administration of sildenafil (50 mg).^47^ Similarly, in 30 older males with ED and a history of ischemic stroke, tadalafil was given orally either as a single 20 mg dose or as 5 mg/day for 7 days, with regional CBF mapped 6 hours later. A mosaic of regional CBF changes was observed after tadalafil, the only pattern being reduced CBF in the perilesional area after treatment.^48^ There was no consistent pattern in regional CBF change between the two PDE5i drugs. As these studies lacked a placebo‐treated control group, it is difficult to interpret them regarding acute effects of PDE5i on CBF in humans.^46^, ^47^, ^48^ In other studies, healthy young adults showed no change in CBF after acute PDE5i treatment.^49^, ^50^

## 5 NEURONAL ACTIONS OF PDE5 INHIBITORS

### 5.1 Animal studies

**FIGURE 4:**
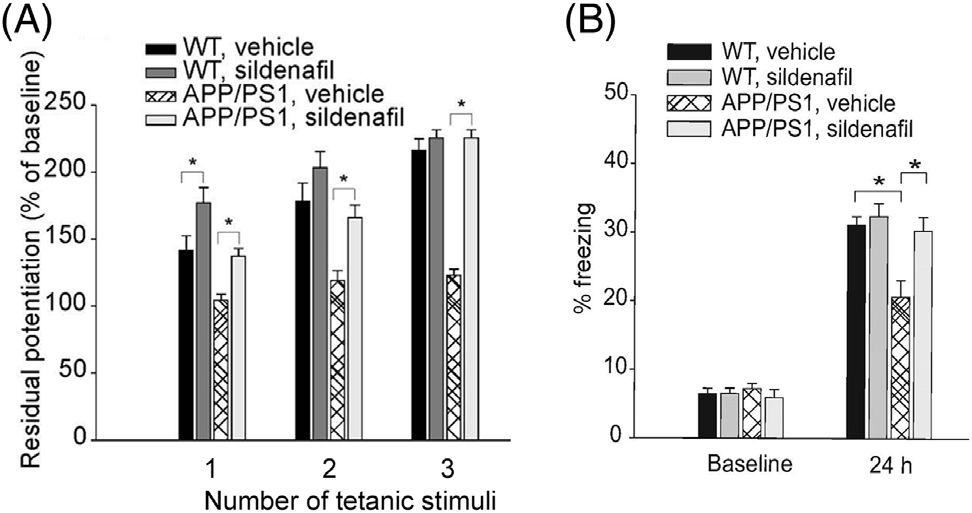
The PDE5i sildenafil augments synaptic strengthening in vitro and in vivo. (A), Sildenafil augments long term potentiation (LTP) in vitro, in hippocampal slices from APP/PS1 transgenic mice. Sildenafil (50 nm) increased LTP in slices from APP/PS1 mice that were potentiated through one or two series of tetanic stimulations (1 tetanus: *P* = 0.007 compared to vehicle‐treated APP/PS1 slices; two tetani: *P* = 0.003; three tetani: *P* < 0.001; *n* = 6 slices from 6 males for each group). Slices from WT mice that received one tetanic stimulation showed a significant increase in LTP with sildenafil treatment, compared to vehicle‐treated WT slices (*P* = 0.048; *n* = 6 slices from 6 males for each group). (B), Sildenafil improves contextual memory in vivo, in 3‐month‐old APP/PS1 transgenic mice. After 24 hours, the reduction in freezing time of APP/PS1 mice is rescued by sildenafil (3 mg/kg intraperitoneally). *n* = 12 (7 males, 5 females) sildenafil‐treated APP/PS1 mice, *n* = 17 (10 males, 7 females) in vehicle‐treated APP/PS1 mice, *P* = 0.013. Sildenafil does not increase freezing in WT littermates (*n* = 17 [10 males, 7 females] vehicle‐treated WT vs. *n* = 14 [8 males, 6 females], sildenafil treated WT, *P* = 0.06). Images reproduced from Puzzo et al.^9^ Copyright [2009] Society for Neuroscience. PDE5i, phosphodiesterase‐5 inhibitor; WT, wild type.

Published data from multiple other laboratories support a cognitive benefit of PDE5i treatment in rats and mice^51^, ^52^, ^53^, ^54^, ^55^, ^56^, ^57^ (examples in Figure 4). In two standard spatial learning paradigms, sildenafil was tested in young male Fischer‐344 rats for cognition‐enhancing efficacy.^15^ In the T‐maze and Morris water maze learning performance was not improved by PDE5i treatment (up to 4.5 mg/kg sildenafil, given 15 minutes before the training session). After a 7‐day drug washout period, PDE5i‐treated animals had better memory retention than aged controls.^15^ Acute treatment with vardenafil also improved memory acquisition in rats, in a cholinergic‐deficit model of amnesia (based on scopolamine treatment).^14^

In mice, a highly potent novel PDE5i KJH‐1002 reversed cognitive impairment, again in a scopolamine‐based model.^16^ Young male mice were given the agent acutely (up to 20 mg/kg, by mouth 15 minutes before testing). Similarly, in young male mice sildenafil reduced noise‐induced cognitive deficits^58^ and vardenafil ameliorated sleep deprivation.^59^ Experiments in transgenic murine models of AD pathology support a cognitive benefit of PDE5i treatment. Young adult APP/PS1 mice, both males and females, were treated with sildenafil (3 mg/kg/day) for 21 days, then underwent behavioral testing 9 weeks later.^9^ Sildenafil improved memory, amyloid plaque load, inflammation, and neurogenesis^9^ (Figure 4). In another AD model, aged J20 mice (age 18 months) of both sexes were treated with sildenafil (15 mg/kg/day) for 10 weeks.^13^ Sildenafil improved memory, tau hyperphosphorylation, and GSK3β phosphorylation,^13^ consistent with mechanistic observations in AD patient iPSC‐derived neurons.^7^ Tadalafil has given more contradictory findings in transgenic mouse models of AD, with some groups observing improved cognitive performance in vivo^13^ and others not.^9^ With exceptions,^9,13^ the majority of rodent studies have selected sildenafil as the PDE5i agent of choice and most have used young, male animals.

In non‐human primates^60^ acute treatment with sildenafil dose‐dependently increased cognitive function, in accord with rodent data. Adult male cynomolgus monkeys (age 5–14 years) received sildenafil (up to 3 mg/kg/day, intramuscularly) every 3 to 4 days, 60 minutes prior to testing. After drug treatment they performed better in a paradigm considered a prefrontal task of executive function (accurate retrieval of a food reward from a small open box).^60^

### 5.2 Human data

Reports of central PDE5i actions in humans are sparse. In young healthy volunteers, acute treatment with vardenafil was tested for central actions on auditory “gating,” an adaptive sensory mechanism whereby the electroencephalogram (EEG) response to a second paired sound stimulus is reduced relative to the first. Vardenafil was administered 90 minutes prior to testing and no effects of the PDE5i on gating were detected.^61^ Several other low‐powered studies of the acute actions of sildenafil on cognitive measures found no effects in stable schizophrenic outpatients (*N* = 17),^62^ or in small cohorts of healthy volunteers (*N* = 6–10).^63^, ^64^ In one of these, changes in scalp EEG were interpreted as evidence of central nervous system penetration by sildenafil.^64^

After more chronic PDE5i treatment, indications of cognitive effects were reported. A cohort of 27 ED patients were treated for 2 months with the novel agent udenafil (100 mg), though unfortunately the study did not include control participants. Significant improvements were noted in variants of the Mini‐Mental State Examination and frontal assessment battery (adapted for Korean language users).^65^

## 6 REAL‐WORLD PERSPECTIVE

### 6.1 The older, multiply medicated neurology patient

Despite recent developments in anti‐amyloid therapies^66^ there remains a large unmet need for drug treatments in dementia. Older patients are frequently under‐represented in clinical trials of anti‐amyloid drugs, due to upper age limits for inclusion and also exclusion of participants with more than trivial cerebrovascular disease. There is a pressing clinical need for therapies that can safely be given to older people with mixed AD and vascular pathologies who, due to demographic aging, will ultimately constitute the majority of people with dementia.

An open question is whether the primary mechanism of action of PDE5i in the brain is via PDE5 activity located within cells of the vasculature (as in ED and PAH) or through direct action on PDE5 located within brain cells. This will determine whether the target patient population should be those with mixed AD and vascular pathology, or whether PDE5i might also be beneficial in people with dementia entirely ascribed to AD pathology, such as sporadic and genetic early‐onset AD, or alternatively to persons with sporadic vascular dementia with no evidence of amyloid deposition, or genetic forms of small vessel disease (such as cerebral autosomal dominant arteriopathy with subcortical infarcts and leukoencephalopathy).

Mechanistic studies in rodents and in vitro platforms support a benefit of PDE5i in AD, and a recent systematic review concluded that a clinical trial of sildenafil for cognitive enhancement in AD is warranted.^17^ This claim was supported by some real‐world big clinical data^7^ but not by others.^34^ The small number of clinical trials that have addressed brain actions of PDE5i treatment have been neutral or shown very modest effects.^36^, ^45^, ^46^, ^67^ These trials are limited by small cohort size,^46^ too brief treatment periods,^36^ and possibly inappropriate disease populations.^67^ They do not preclude future trials to test for a disease‐modifying effect on AD or vascular pathology, or a symptomatic effect in AD.

### 6.2 Future directions

The results discussed in Section 5 raise multiple questions for drug development in VCID. Do in vitro and in vivo preclinical paradigms provide non‐translatable findings because these systems do not adequately model the complexity of vascular and AD pathologies in an aging human body? Does the duration of clinical trials with drugs that would target one disease component suffice to reach statistically significant effects on cognition, given the multi‐factorial nature of such an outcome and evolution of pathologies over many years prior to symptom onset? Specifically, are PDE5i targeting AD‐related neuropathology or brain vascular aging? What should be emphasized in the design of a definitive randomized clinical trial for this class of therapeutics and what would be the interventional window in which effect could be maximized? Finally, what biomarker sets could be used to respond to these questions and design a definitive clinical trial to determine whether PDE5i drugs have a place in the dementia pharmaceutical cabinet, for prevention or treatment.

Prior to embarking on future clinical trials of PDE5i in ADRD, several translational directions are indicated. For sildenafil and other agents currently licensed for clinical use, definitive data on brain penetration in older humans is a clear need. Repurposing these existing medicines will bring its own challenges, around commercialization, for example, though these are tractable.^68^ In terms of bringing on novel drugs, the design and syntheses of new small molecules with high BBB permeability and longer half‐life (≈24 hours) would be optimal, with high specificity for PDE5 (over PDE6 and PDE11 in particular).

## 7 SYNTHESIS AND CONCLUSIONS

In our view PDE5 inhibitors merit further investigation for possible use as dementia preventative treatments, with relevance to ADRD and VCID. We believe it is a tenable hypothesis that brain‐penetrant PDE5i drugs can augment NO‐driven synaptic strengthening and so improve memory function. Testing this will require chronic treatment with a PDE5i (for at least 12 months) in a placebo‐controlled, randomized clinical trial. Such a trial should recruit both female and male participants, including patients aged ≥80 years, with a long‐term follow‐up. The choice of drug for future clinical trials will be informed by multiple considerations including tolerability in older people, brain penetration, and plasma half‐life.

## AUTHOR CONTRIBUTIONS

Conceptualization: Atticus H. Hainsworth. Ideas: all authors. Writing original draft: Atticus H. Hainsworth. Writing—review & editing: all authors.

## CONFLICT OF INTEREST STATEMENT

Dr. Hainsworth has received honoraria from Eli‐Lilly and from NIA. He is chair of the Dementias Platform UK Vascular Experimental Medicine group. Dr. Arancio is a co‐inventor of a series of PDE5 inhibitors that were licensed by Columbia University to Aribio Co. Dr. Elahi is chair of the Vascular Cognitive Disorders Group within ISTAART. Dr. Isaacs has received advisory board fees from Roche and Nestle Scientific, consultancy fees from Roche, and a speaker's fee from Biogen, all paid to his institution. He has received funded conference registration, travel, and accommodation from Roche. Dr. Cheng has received honoraria from National Institute on Aging (NIA).

## DISCLOSURES

The views expressed in this paper are those of the individual authors. FC, FME, AHH, and JDI are members of The Alzheimer's Association International Society to Advance Alzheimer's Research and Treatment (ISTAART). This Perspective arose from a Featured Research Session at AAIC2022.

## CONSENT STATEMENT

All human subjects provided informed consent.

## Supporting information




Supplementary information: ICMJE DISCLOSURE FORM

## References

[1] Corriveau RA , Bosetti F , Emr M , et al. The science of vascular contributions to cognitive impairment and dementia (VCID): a framework for advancing research priorities in the cerebrovascular biology of cognitive decline. Cell Mol Neurobiol. 2016;36:281‐288. 27095366 10.1007/s10571-016-0334-7 PMC4859348

[2] Gorelick PB , Scuteri A , Black SE , et al. Vascular contributions to cognitive impairment and dementia: a statement for healthcare professionals from the American heart association/American stroke association. Stroke. 2011;42:2672‐2713. 21778438 10.1161/STR.0b013e3182299496 PMC3778669

[3] Hainsworth AH , Elahi FM , Corriveau RA . An introduction to therapeutic approaches to vascular cognitive impairment. Cereb Circ Cogn Behav. 2021;2:100033. 34950896 10.1016/j.cccb.2021.100033 PMC8661126

[4] Smith EE , Barber P , Field TS , et al. Canadian consensus conference on diagnosis and treatment of dementia (CCCDTD)5: guidelines for management of vascular cognitive impairment. Alzheimers Dement (N Y). 2020;6:e12056. 33209971 10.1002/trc2.12056 PMC7657196

[5] Fang J , Pieper AA , Nussinov R , et al. Harnessing endophenotypes and network medicine for Alzheimer's drug repurposing. Med Res Rev. 2020;40:2386‐426. 32656864 10.1002/med.21709 PMC7561446

[6] Conti M , Beavo J . Biochemistry and physiology of cyclic nucleotide phosphodiesterases: essential components in cyclic nucleotide signaling. Annu Rev Biochem 2007;76:481‐511. 17376027 10.1146/annurev.biochem.76.060305.150444

[7] Fang J , Zhang P , Zhou Y , et al. Endophenotype‐based in silico network medicine discovery combined with insurance record data mining identifies sildenafil as a candidate drug for Alzheimer's disease. Nature Aging. 2021;1:1175‐1188. 35572351 10.1038/s43587-021-00138-z PMC9097949

[8] Fiorito J , Saeed F , Zhang H , et al. Synthesis of quinoline derivatives: discovery of a potent and selective phosphodiesterase 5 inhibitor for the treatment of Alzheimer's disease. Eur J Med Chem. 2013;60:285‐294. 23313637 10.1016/j.ejmech.2012.12.009 PMC3582828

[9] Puzzo D , Staniszewski A , Deng SX , et al. Phosphodiesterase 5 inhibition improves synaptic function, memory, and amyloid‐beta load in an Alzheimer's disease mouse model. J Neurosci 2009;29:8075‐8086. 19553447 10.1523/JNEUROSCI.0864-09.2009 PMC6666028

[10] Puzzo D , Vitolo O , Trinchese F , Jacob JP , Palmeri A , Arancio O . Amyloid‐beta peptide inhibits activation of the nitric oxide/cGMP/cAMP‐responsive element‐binding protein pathway during hippocampal synaptic plasticity. J Neurosci. 2005;25:6887‐6897. 16033898 10.1523/JNEUROSCI.5291-04.2005 PMC6725343

[11] Acquarone E , Argyrousi EK , van Den Berg M , et al. Synaptic and memory dysfunction induced by tau oligomers is rescued by up‐regulation of the nitric oxide cascade. Mol Neurodegener. 2019;14:26. 31248451 10.1186/s13024-019-0326-4 PMC6598340

[12] Zuccarello E , Acquarone E , Calcagno E , et al. Development of novel phosphodiesterase 5 inhibitors for the therapy of Alzheimer's disease. Biochem Pharmacol. 2020;176:113818. 31978378 10.1016/j.bcp.2020.113818 PMC7263960

[13] Garcia‐Barroso C , Ricobaraza A , Pascual‐Lucas M , et al. Tadalafil crosses the blood‐brain barrier and reverses cognitive dysfunction in a mouse model of AD. Neuropharmacology. 2013;64:114‐123. 22776546 10.1016/j.neuropharm.2012.06.052

[14] Akkerman S , Blokland A , van Goethem NP , et al. PDE5 inhibition improves acquisition processes after learning via a central mechanism. Neuropharmacology. 2015;97:233‐239. 26027948 10.1016/j.neuropharm.2015.04.019

[15] Devan BD , Bowker JL , Duffy KB , et al. Phosphodiesterase inhibition by sildenafil citrate attenuates a maze learning impairment in rats induced by nitric oxide synthase inhibition. Psychopharmacology (Berl). 2006;183:439‐445. 16320087 10.1007/s00213-005-0232-z

[16] Zhang L , Seo JH , Li H , Nam G , Yang HO . The phosphodiesterase 5 inhibitor, KJH‐1002, reverses a mouse model of amnesia by activating a cGMP/cAMP response element binding protein pathway and decreasing oxidative damage. Br J Pharmacol. 2018;175:3347‐3360. 29847860 10.1111/bph.14377 PMC6057906

[17] Sanders O . SildeNAFIL FOR THE TREATMENT OF Alzheimer's Disease: a systematic review. J Alzheimers Dis Rep. 2020;4:91‐106. 32467879 10.3233/ADR-200166 PMC7242821

[18] Sanderson TM , Sher E . The role of phosphodiesterases in hippocampal synaptic plasticity. Neuropharmacology. 2013;74:86‐95. 23357335 10.1016/j.neuropharm.2013.01.011

[19] Lakics V , Karran EH , Boess FG . Quantitative comparison of phosphodiesterase mRNA distribution in human brain and peripheral tissues. Neuropharmacology. 2010;59:367‐374. 20493887 10.1016/j.neuropharm.2010.05.004

[20] Teich AF , Sakurai M , Patel M , et al. PDE5 exists in human neurons and is a viable therapeutic target for neurologic disease. J Alzheimers Dis. 2016;52:295‐302. 26967220 10.3233/JAD-151104 PMC4927884

[21] Vasita E , Yasmeen S , Andoh J , et al. The cGMP‐degrading enzyme phosphodiesterase‐5 (PDE5) in cerebral small arteries of older people. J Neuropathol Exp Neurol. 2019;78:191‐194. 30590671 10.1093/jnen/nly117

[22] Gibson A . Phosphodiesterase 5 inhibitors and nitrergic transmission‐from zaprinast to sildenafil. Eur J Pharmacol. 2001;411:1‐10. 11137852 10.1016/s0014-2999(00)00824-4

[23] Goldstein I , Lue TF , Padma‐Nathan H , Rosen RC , Steers WD , Wicker PA . Oral sildenafil in the treatment of erectile dysfunction. Sildenafil Study Group. N Engl J Med. 1998;338:1397‐1404. 9580646 10.1056/NEJM199805143382001

[24] Hong JH , Kwon YS , Kim IY . Pharmacodynamics, pharmacokinetics and clinical efficacy of phosphodiesterase‐5 inhibitors. Expert Opin Drug Metab Toxicol. 2017;13:183‐192. 27690667 10.1080/17425255.2017.1244265

[25] Blount MA , Beasley A , Zoraghi R , et al. Binding of tritiated sildenafil, tadalafil, or vardenafil to the phosphodiesterase‐5 catalytic site displays potency, specificity, heterogeneity, and cGMP stimulation. Mol Pharmacol. 2004;66:144‐152. 15213306 10.1124/mol.66.1.144

[26] Card GL , England BP , Suzuki Y , et al. Structural basis for the activity of drugs that inhibit phosphodiesterases. Structure. 2004;12:2233‐2247. 15576036 10.1016/j.str.2004.10.004

[27] Mancina R , Filippi S , Marini M , et al. Expression and functional activity of phosphodiesterase type 5 in human and rabbit vas deferens. Mol Hum Reprod. 2005;11:107‐115. 15640438 10.1093/molehr/gah143

[28] Soderling SH , Bayuga SJ , Beavo JA . Identification and characterization of a novel family of cyclic nucleotide phosphodiesterases. J Biol Chem. 1998;273:15553‐15558. 9624145 10.1074/jbc.273.25.15553

[29] Cahill KB , Quade JH , Carleton KL , Cote RH . Identification of amino acid residues responsible for the selectivity of tadalafil binding to two closely related phosphodiesterases, PDE5 and PDE6. J Biol Chem. 2012;287:41406‐41416. 23033484 10.1074/jbc.M112.389189 PMC3510839

[30] Weeks JL , Zoraghi R , Beasley A , Sekhar KR , Francis SH , Corbin JD . High biochemical selectivity of tadalafil, sildenafil and vardenafil for human phosphodiesterase 5A1 (PDE5) over PDE11A4 suggests the absence of PDE11A4 cross‐reaction in patients. Int J Impot Res. 2005;17:5‐9. 15538396 10.1038/sj.ijir.3901283

[31] Doh H , Shin CY , Son M , et al. Mechanism of erectogenic effect of the selective phosphodiesterase type 5 inhibitor, DA‐8159. Arch Pharm Res. 2002;25:873‐878. 12510841 10.1007/BF02977007

[32] Fiorito J , Vendome J , Saeed F , et al. Identification of a novel 1,2,3,4‐Tetrahydrobenzo[b][1,6]naphthyridine analogue as a potent phosphodiesterase 5 inhibitor with improved aqueous solubility for the treatment of Alzheimer's disease. J Med Chem. 2017;60:8858‐8875. 28985058 10.1021/acs.jmedchem.7b00979 PMC9614283

[33] Charpignon ML , Vakulenko‐Lagun B , Zheng B , et al. Causal inference in medical records and complementary systems pharmacology for metformin drug repurposing towards dementia. Nat Commun. 2022;13:7652. 36496454 10.1038/s41467-022-35157-w PMC9741618

[34] Desai RJ , Mahesri M , Lee SB , et al. No association between initiation of phosphodiesterase‐5 inhibitors and risk of incident Alzheimer's disease and related dementia: results from the Drug Repurposing for Effective Alzheimer's Medicines study. Brain Commun. 2022;4:fcac247. 36330433 10.1093/braincomms/fcac247 PMC9598543

[35] Bon CL , Garthwaite J . On the role of nitric oxide in hippocampal long‐term potentiation. J Neurosci. 2003;23:1941‐1948. 12629199 10.1523/JNEUROSCI.23-05-01941.2003 PMC6741944

[36] Pauls MM , Binnie LR , Benjamin P , et al. The PASTIS trial. Testing tadalafil for possible use in vascular cognitive impairment Alzheimers & Dementia. Alzheimers Dement. 2022;18(12):2393‐2402. 35135037 10.1002/alz.12559 PMC10078742

[37] Cannistraro RJ , Badi M , Eidelman BH , Dickson DW , Middlebrooks EH , Meschia JF . CNS small vessel disease: a clinical review. Neurology. 2019;92:1146‐1156. 31142635 10.1212/WNL.0000000000007654 PMC6598791

[38] Pauls MMH , Clarke N , Trippier S , et al. Perfusion by arterial spin labelling following single dose tadalafil in small vessel disease (PASTIS): study protocol for a randomised controlled trial. Trials. 2017;18:229. 28532471 10.1186/s13063-017-1973-9 PMC5440904

[39] Forgue ST , Patterson BE , Bedding AW , et al. Tadalafil pharmacokinetics in healthy subjects. Br J Clin Pharmacol. 2006;61:280‐288. 16487221 10.1111/j.1365-2125.2005.02553.x PMC1885023

[40] Forgue ST , Phillips DL , Bedding AW , et al. Effects of gender, age, diabetes mellitus and renal and hepatic impairment on tadalafil pharmacokinetics. Br. J. Clin. Pharmacol. 2007;63:24‐35. 16869816 10.1111/j.1365-2125.2006.02726.x PMC2000708

[41] Unceta N , Echeazarra L , Montana M , et al. Validation of an LC‐ESI‐MS/MS method for the quantitation of phosphodiesterase‐5 inhibitors and their main metabolites in rat serum and brain tissue samples. J Pharm Biomed Anal. 2012;70:529‐533. 22647499 10.1016/j.jpba.2012.04.030

[42] Garcia‐Barroso C , Ugarte A , Martinez M , et al. Phosphodiesterase inhibition in cognitive decline. J Alzheimers Dis. 2014;42(Suppl 4):S561‐S573. 25125473 10.3233/JAD-141341

[43] Gomez‐Vallejo V , Ugarte A , Garcia‐Barroso C , et al. Pharmacokinetic investigation of sildenafil using positron emission tomography and determination of its effect on cerebrospinal fluid cGMP levels. J Neurochem. 2016;136:403‐15. 26641206 10.1111/jnc.13454

[44] Alber J , Alladi S , Bae HJ , et al. White matter hyperintensities in vascular contributions to cognitive impairment and dementia (VCID): Knowledge gaps and opportunities. Alzheimers Dement (N Y). 2019;5:107‐117. 31011621 10.1016/j.trci.2019.02.001 PMC6461571

[45] Samudra N , Motes M , Lu H , et al. A pilot study of changes in medial temporal lobe fractional amplitude of low frequency fluctuations after sildenafil administration in patients with Alzheimer's Disease. J Alzheimers Dis. 2019;70:163‐170. 31156166 10.3233/JAD-190128 PMC6743329

[46] Sheng M , Lu H , Liu P , et al. Sildenafil improves vascular and metabolic function in patients with Alzheimer's Disease. J Alzheimers Dis. 2017;60:1351‐1364. 29036811 10.3233/JAD-161006 PMC5805465

[47] Lorberboym M , Mena I , Wainstein J , Boaz M , Lampl Y . The effect of sildenafil citrate (Viagra) on cerebral blood flow in patients with cerebrovascular risk factors. Acta Neurol Scand. 2010;121:370‐376. 20028342 10.1111/j.1600-0404.2009.01307.x

[48] Lorberboym M , Makhline E , Lampl Y . Regional cerebral blood flow following single‐dose and continuous‐dose tadalafil after stroke. Acta Neurol Scand. 2014;130:380‐386. 25208597 10.1111/ane.12279

[49] Jahshan S , Dayan L , Jacob G . Nitric oxide‐sensitive guanylyl cyclase signaling affects CO2‐dependent but not pressure‐dependent regulation of cerebral blood flow. Am J Physiol Regul Integr Comp Physiol. 2017;312:R948‐R955. 28356297 10.1152/ajpregu.00241.2016

[50] Kruuse C , Hansen AE , Larsson HB , Lauritzen M , Rostrup E . Cerebral haemodynamic response or excitability is not affected by sildenafil. J Cereb Blood Flow Metab. 2009;29:830‐839. 19209179 10.1038/jcbfm.2009.10

[51] Baratti CM , Boccia MM . Effects of sildenafil on long‐term retention of an inhibitory avoidance response in mice. Behav Pharmacol. 1999;10:731‐7. 10780288 10.1097/00008877-199912000-00004

[52] Boccia MM , Blake MG , Krawczyk MC , Baratti CM . Sildenafil, a selective phosphodiesterase type 5 inhibitor, enhances memory reconsolidation of an inhibitory avoidance task in mice. Behav Brain Res. 2011;220:319‐324. 21333692 10.1016/j.bbr.2011.02.016

[53] Rodefer JS , Saland SK , Eckrich SJ . Selective phosphodiesterase inhibitors improve performance on the ED/ID cognitive task in rats. Neuropharmacology. 2012;62:1182‐90. 21856317 10.1016/j.neuropharm.2011.08.008

[54] Cuadrado‐Tejedor M , Hervias I , Ricobaraza A , et al. Sildenafil restores cognitive function without affecting beta‐amyloid burden in a mouse model of Alzheimer's disease. Br J Pharmacol. 2011;164:2029‐2041. 21627640 10.1111/j.1476-5381.2011.01517.x PMC3246665

[55] Orejana L , Barros‐Minones L , Jordan J , Puerta E , Aguirre N . Sildenafil ameliorates cognitive deficits and tau pathology in a senescence‐accelerated mouse model. Neurobiol Aging. 2012;33:625 e11‐20. 10.1016/j.neurobiolaging.2011.03.018 21546125

[56] Rutten K , Vente JD , Sik A , Ittersum MM , Prickaerts J , Blokland A . The selective PDE5 inhibitor, sildenafil, improves object memory in Swiss mice and increases cGMP levels in hippocampal slices. Behav Brain Res. 2005;164:11‐16. 16076505 10.1016/j.bbr.2005.04.021

[57] Prickaerts J , van Staveren WC , Sik A , et al. Effects of two selective phosphodiesterase type 5 inhibitors, sildenafil and vardenafil, on object recognition memory and hippocampal cyclic GMP levels in the rat. Neuroscience. 2002;113:351‐361. 12127092 10.1016/s0306-4522(02)00199-9

[58] Sikandaner HE , Park SY , Kim MJ , Park SN , Yang DW . Neuroprotective effects of sildenafil against oxidative stress and memory dysfunction in mice exposed to noise stress. Behav Brain Res. 2017;319:37‐47. 27836585 10.1016/j.bbr.2016.10.046

[59] Heckman PRA , Roig Kuhn F , Raven F , et al. Phosphodiesterase inhibitors roflumilast and vardenafil prevent sleep deprivation‐induced deficits in spatial pattern separation. Synapse. 2020;74:e22150. 32056276 10.1002/syn.22150 PMC9285343

[60] Rutten K , Basile JL , Prickaerts J , Blokland A , Vivian JA . Selective PDE inhibitors rolipram and sildenafil improve object retrieval performance in adult cynomolgus macaques. Psychopharmacology (Berl). 2008;196:643‐648. 18034336 10.1007/s00213-007-0999-1 PMC2244695

[61] Reneerkens OA , Sambeth A , Van Duinen MA , Blokland A , Steinbusch HW , Prickaerts J . The PDE5 inhibitor vardenafil does not affect auditory sensory gating in rats and humans. Psychopharmacology (Berl). 2013;225:303‐312. 22855271 10.1007/s00213-012-2817-7

[62] Goff DC , Cather C , Freudenreich O , et al. A placebo‐controlled study of sildenafil effects on cognition in schizophrenia. Psychopharmacology (Berl). 2009;202:411‐417. 18716761 10.1007/s00213-008-1278-5 PMC2704618

[63] Grass H , Klotz T , Fathian‐Sabet B , Berghaus G , Engelmann U , Kaferstein H . Sildenafil (Viagra): is there an influence on psychological performance? Int Urol Nephrol. 2001;32:409‐412. 11583362 10.1023/a:1017573722074

[64] Schultheiss D , Muller SV , Nager W , et al. Central effects of sildenafil (Viagra) on auditory selective attention and verbal recognition memory in humans: a study with event‐related brain potentials. World J Urol. 2001;19:46‐50. 11289570 10.1007/pl00007092

[65] Shim YS , Pae CU , Kim SW , Kim HW , Kim JC , Koh JS . Effects of repeated dosing with Udenafil (Zydena) on cognition, somatization and erection in patients with erectile dysfunction: a pilot study. Int J Impot Res. 2011;23:109‐114. 21544084 10.1038/ijir.2011.13

[66] van Dyck CH , Swanson CJ , Aisen P , et al. Lecanemab in early Alzheimer's Disease. N Engl J Med. 2023;388:9‐21. 36449413 10.1056/NEJMoa2212948

[67] Di Cesare F , Mancuso J , Woodward P , Bednar MM , Loudon PT , Group ASS . Phosphodiesterase‐5 inhibitor PF‐03049423 effect on stroke recovery: a double‐blind, placebo‐controlled randomized clinical trial. J Stroke Cerebrovasc Dis. 2016;25:642‐649. 26738812 10.1016/j.jstrokecerebrovasdis.2015.11.026

[68] Shineman DW , Alam J , Anderson M , et al. Overcoming obstacles to repurposing for neurodegenerative disease. Ann Clin Transl Neurol. 2014;1:512‐518. 25356422 10.1002/acn3.76 PMC4184781

